# Airway Basal Stem Cell Population Is Enlarged in Bronchial Thermoplasty Treated Airways in Severe Asthma Patients

**DOI:** 10.1111/cea.70071

**Published:** 2025-05-04

**Authors:** Tessa E. Gillett, Sofi M. Vassileva, Els Weersink, Jouke Annema, René Lutter, Martijn C. Nawijn, Maarten van den Berge, Peter I. Bonta, Abilash Ravi

**Affiliations:** ^1^ Experimental Pulmonary and Inflammatory Research, Department of Pathology and Medical Biology University Medical Center Groningen, University of Groningen Groningen the Netherlands; ^2^ Groningen Research Institute for Asthma and COPD University Medical Center Groningen, University of Groningen Groningen the Netherlands; ^3^ Department of Respiratory Medicine Amsterdam University Medical Center, University of Amsterdam Amsterdam the Netherlands; ^4^ Department of Pulmonology University Medical Center Groningen, University of Groningen Groningen the Netherlands

**Keywords:** asthma, basal cells, bronchial thermoplasty, clinical immunology, deconvolution, epithelium, omics and systems biology, quality of life

**Summary**

- Deconvolution analysis predicts an enlarged basal cell population in bronchial thermoplasty‐treated airways.
- Increased basal cells may contribute to clinical improvement post treatment in severe asthma patients.

To the Editor,

Approximately 5% of asthma patients have severe disease, not well‐controlled [1] and are treated using biologicals and/or bronchial thermoplasty (BT) [2]. BT applies temperature‐controlled radio‐frequency energy to medium‐sized and larger airways to impact airway remodelling, including reduction of airway smooth muscle and changes in extra‐cellular matrix, leading to improved asthma‐related quality of life and reduced exacerbations [3]. We previously demonstrated that BT alters metabolism [4] and reduces inflammation [5] in bronchial brushes subjected to RNA‐sequencing. During epithelial injury, basal cells can self‐renew, differentiate, and drive immune responses along with macrophages and dendritic cells. BT‐induced changes in the cell type composition of the bronchial epithelium are unknown. To investigate this, we performed cell type deconvolution on bulk RNA‐sequencing data from bronchial brushes collected in the TASMA study [6].

The TASMA study performed an extensive clinical characterisation and samples were collected before and 6 months after BT, in treated airways and at 6 months in the untreated right middle lobe, but not before from the same subjects. Bronchial brushes were obtained from severe asthma patients (*n* = 23) in the segmental and subsegmental bronchi and contain sub‐types of bronchial epithelium, along with alveolar macrophages, dendritic cells and lymphocytes. The TASMA study design, inclusion and exclusion criteria and methods have been previously described [6]. The study was approved by the Medical Ethics Committee and all subjects gave their written informed consent (NL45394.018.13) [6]. Cell type deconvolution was performed with CIBERSORTx, using single cell RNA‐sequencing data of bronchial brushes from the Human Lung Cell Atlas as a reference. Accuracy was validated by deconvolution of HLCA‐derived bronchial brush pseudobulk samples of known cell type composition. Additional information on patient characteristics, sample collection, RNA isolation, sequencing and deconvolution analysis are available in the following repository: https://zenodo.org/records/14857109.

Deconvolution analysis estimated proportions of basal (15.49%), ciliated lineage (ciliated and deuterosomal cells;79.47%), secretory (0.15%), and rare (ionocytes, neuroendocrine and tuft cells;0.24%) epithelial cells, monocytes (0.87%), alveolar (1.78%) and luminal macrophages (0.25%), dendritic cells (1.47%), mast cells (0.23%), B‐cell lineage cells (B‐ and plasma cells;0.05%), and T‐, NK and innate lymphoid cells (0.06%) in bronchial brushes of all samples (Figure 1A). We excluded cell types with an estimated proportion of zero in more than 60% of the samples from further statistical analyses. Six months after BT, we found estimated basal cell proportions to be significantly higher in treated compared to untreated regions, from the same patients, whereas the proportions of alveolar macrophages, ciliated lineage cells, and dendritic cells did not differ significantly (Figure 1B). When comparing brushes from the treated lobe obtained before and 6 months after BT, none of the deconvoluted cell types were significantly different (data not shown). This may be partly explained by variability over the 6‐month period, e.g., due to seasonal influences and intercurrent infections, which is eliminated by sampling treated and untreated regions at the same time point.

**FIGURE 1:**
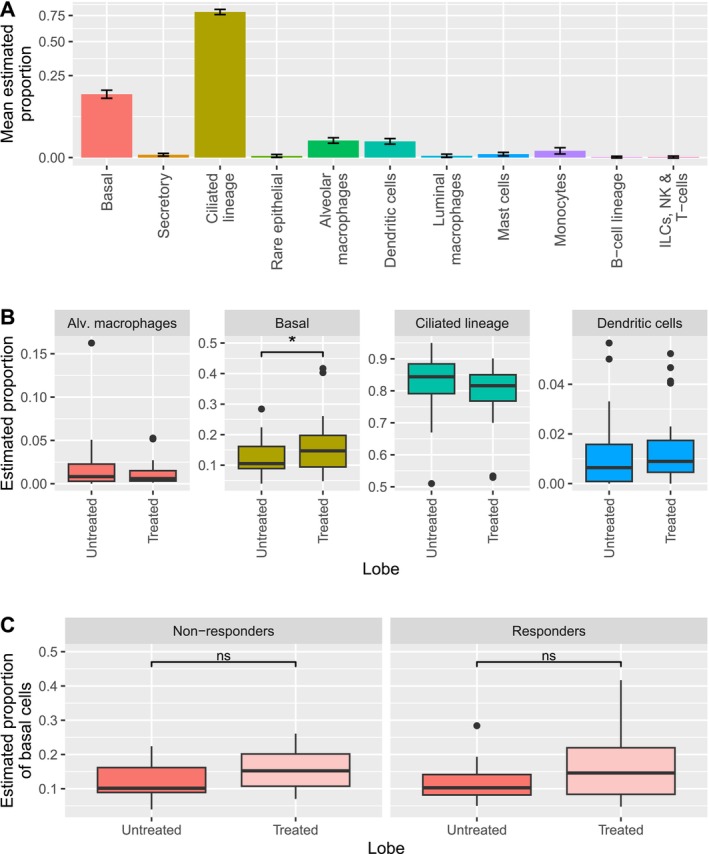
Deconvolution analysis of bronchial brushes obtained before and 6 months after bronchial thermoplasty treatment (BT). (A) Average estimated proportion of each cell type across all samples (*n* = 23), obtained from the treated lobe prior to BT, from the untreated lobe 6 months after BT, and from the treated lobe 6 months after BT; error bars indicate standard error of the mean (±SEM). (B) Estimated proportions of alveolar macrophages, basal cells, ciliated lineage cells, and dendritic cells comparing untreated and the treated lobe 6 months after BT. (C) Estimated proportions of basal cells in responders (*n* = 11) and non‐responders (*n* = 12) in the untreated versus treated lobe, 6 months after BT. Analysis of differences was conducted using paired two‐tailed *t*‐test, and differences of *p* value < 0.05 are considered significant, and error bars indicate standard deviation (±SD).

We previously showed that not all severe asthma patients respond to BT treatment, as defined by a clinically relevant improvement in Asthma Quality of Life Questionnaire score of > 0.5 [6]. The brushes obtained from 23 patients analysed in Figure 1B were separated as responders (*n* = 11) and non‐responders (*n* = 12). When comparing estimated basal cell proportions in treated versus untreated regions at 6 months after BT, no difference was found between BT responders and non‐responders (Figure 1C). Similarly, ciliated lineage cells, alveolar macrophages, and dendritic cells were not significantly different between the two groups (data not shown). To assess proliferation activity of bronchial cells in the treated compared to untreated regions, we analysed the expression of proliferation marker genes *PCNA, TOP2A, MKI67* and *MCM2*. No significant differences were observed (data not shown).

In this study, we show a higher proportion of basal cells in BT‐treated regions, which have the capacity to self‐renew, transition to committed progenitors, and later differentiate into secretory and ciliated cells [7]. A recent study has shown airway basal cell transplantation's potential as a cell‐based therapy for airway epithelial cell damage, illustrating the importance of replenishing basal cells to promote repair and regeneration [8]. Importantly, we validated the signature matrix accuracy; also, estimated cell type proportions were comparable to the proportions identified previously in a single cell (sc)RNA‐sequencing dataset of bronchial brushes obtained from asthma patients for another study [9]. Limitations in this study include the lack of bronchial brushes from the untreated right middle lobe at baseline for comparison of changes in cell populations over time in the untreated regions. Therefore, we cannot exclude that changes in the untreated middle lobe over 6 months could have contributed to the observed higher number of basal cells. However, we were able to compare brushes obtained from treated and untreated regions at the same time point, removing the effects of variation over time. No differences were observed between responders and non‐responders to BT treatment, which may be due to limited sample size. In this deconvolution analysis, the functional and activation state of the cell types analysed could not be determined. Additional scRNA‐sequencing analysis of subtypes of basal cells and mesenchymal cell populations in epithelial brushes and airway biopsies would provide insights into the impact of BT on airway repair and remodelling and/or inflammation. Furthermore, analysis of changes in epithelial populations at earlier time points after BT could be interesting to study effects over time.

In conclusion, using cell type deconvolution, we show an enlarged basal cell population in bronchial brushes of patients with severe asthma in BT‐treated airways compared to untreated airways, which may potentially contribute to the clinical improvement following BT.

## Author Contributions

The author takes full responsibility for this article.

## Conflicts of Interest

The authors declare no conflicts of interest.

## Data Availability

The data that support the findings of this study are available from the corresponding author upon reasonable request.
